# Geospatial mapping of disparities in out-of-hospital cardiac arrests in the Swiss canton of Fribourg, 2018–2022: A retrospective observational study

**DOI:** 10.1016/j.resplu.2025.101075

**Published:** 2025-08-23

**Authors:** Cynthia Gay, Ludovic Galofaro, Théophile Emmanouilidis, Diane Blaser, Sébastien Pugnale, Dorian Garin, Alexis Cogne, Vincent Ribordy, Youcef Guechi

**Affiliations:** a Faculty of Science and Medicine, University of Fribourg, 20 Avenue de l’Europe, 1700 Fribourg, Switzerland; b Department of Emergency Medicine, HFR Fribourg University and Teaching Hospital, 2/6 Chemin des Pensionnats, 1752 Villars-sur-Glâne, Switzerland; c Geoinformation Service of the Canton of Fribourg, 13 Rue Joseph-Piller, 1700 Fribourg, Switzerland; d Education and Health Promotion Laboratory, Sorbonne Paris Nord University, Villetaneuse, France; e Department of Anesthesiology and Intensive Care Medicine, SZO Visp, 3930 Switzerland; f Department of Cardiology, HFR Fribourg University and Teaching Hospital, 2/6 Chemin des Pensionnats, 1752 Villars-sur-Glâne, Switzerland; g Ecole Polytechnique Fédérale de Lausanne, Rte cantonale, 1015 Lausanne, Switzerland

**Keywords:** OHCA, Return to spontaneous circulation, Geographic information systems, Geospatial dependence, Incidence, Survival

## Abstract

**Background:**

Out-of-hospital cardiac arrest (OHCA) has a high mortality rate worldwide. A first responder (FR) and automated external defibrillator (AED) network was implemented to complement emergency medical services (EMS) in the Swiss canton of Fribourg. This study aims to assess geospatial disparities in FR deployment, AED usage and prehospital response efficiency relative to OHCA clusters.

**Methods:**

This retrospective observational study analysed all OHCA cases recorded in the Swiss Registry of Cardiac Arrest between 2018 and 2022, which occurred in the canton of Fribourg. We used visual spatial mapping to illustrate clusters of OHCA survival and explore their relationship with FR presence and AED use, including outcome proportions in five predefined geographical zones. Multivariate exact logistic regression models were constructed to assess the impact of the five geographical zones in which OHCA occurred on survival to hospital discharge.

**Results:**

Of 1127 OHCA included, 34 % had a FR on-site and an AED was used in 19 % of cases. All OHCA clusters corresponded to the most densely inhabited areas. Survival rates were highest in urban areas (8.5 %) but decreased to 3.6 % in sparsely populated zones. Cardiopulmonary resuscitation (CPR) performance and AED use by first responders or bystanders showed no statistically significant impact across geographic areas. Heatmaps of FR deployment showed a lower intensity in urban areas and a more even distribution across the territory. Despite a higher AED density in urban areas, usage remained low (12 %). By the end of 2022, 2050 FRs and 549 AEDs were registered in the canton, which remains below international recommendations.

**Conclusion:**

Geospatial disparities highlighted the need for optimized FR recruitment, improved AED distribution and refined EMS activation strategies to enhance OHCA survival rates. These findings provide actionable insights for targeted resource allocation of the existing system at the cantonal level.

## Introduction

Out-of-hospital cardiac arrest (OHCA) has a high mortality rate of approximately 90 %,^1^ with an estimated incidence of 84/100,000 inhabitants in Europe.^2^, ^3^ Providing early cardiopulmonary resuscitation (CPR) could double or even quadruple survival, while longer defibrillation time decreases survival by 10–12 % per minute.^4^ In this context, mobile technology is strongly supported by guidelines^5^, ^6^, ^7^ helping first responder (FR) network systems to increase bystander CPR and automated external defibrillator (AED) use, thus improving the survival rate.^8^, ^9^, ^10^, ^11^, ^12^, ^13^ AED distribution has been explored and guidelines suggest that it should be within 100 m of OHCA events to maximise coverage, but their optimal placement remains a subject of debate.^4^, ^14^ Therefore the geographical distribution of these resources plays a crucial role in ensuring rapid response times and improving outcomes for patients.^15^, ^16^, ^17^, ^18^, ^19^, ^20^

Geospatial analysis helps to optimise resource allocation^16^, ^21^, ^22^ as variations in OHCA incidence are influenced by socioeconomic factors.^23^, ^24^, ^25^, ^26^, ^27^, ^28^ In Switzerland, the Swiss Registry of Cardiac Arrest (SWISSRECA) collects data using standardized Utstein criteria.^29^ The canton of Ticino implemented a comprehensive OHCA management system involving trained lay persons^6^, ^30^, ^31^, ^32^ and a significant number of AEDs in relation to the population, leading to improved outcomes.^33^ Recent studies in Ticino and other Swiss cantons have focused on mapping OHCA risks and survival rates, underscoring the need for a strategic AED and FR network deployment.^26^, ^34^, ^35^ However, studies in cantons such as Vaud and Ticino have also revealed gaps in resource accessibility, emphasizing the importance of geospatial assessment.^25^, ^35^, ^36^

In the canton of Fribourg, a recent study investigating the FR impact highlighted that the presence of a FR did not significantly improve OHCA survival. The study suggested that there could be a lack of FR, AED and emergency medical services (EMS) territorial coverage.^37^ To date, there has been no analysis of the entire OHCA prehospital response system in Fribourg. With this study, we sought to evaluate the geospatial repartition of OHCA and effectiveness of the cantonal prehospital response system. The primary objective was to identify spatial clusters and compare them to the population distribution. Secondary objectives focused on analysing survival and mortality clusters, considering the presence of FR and AED use. Finally, we aimed to determine the proportion of survival, FR presence and AED use within five strategic zones: urban areas; intermediate areas; rural areas; a ‘FR Plus’ zone (FRs with advanced training); and regions with EMS response times exceeding 10 min.

## Methods

### Study design and population

**Fig. 1:**
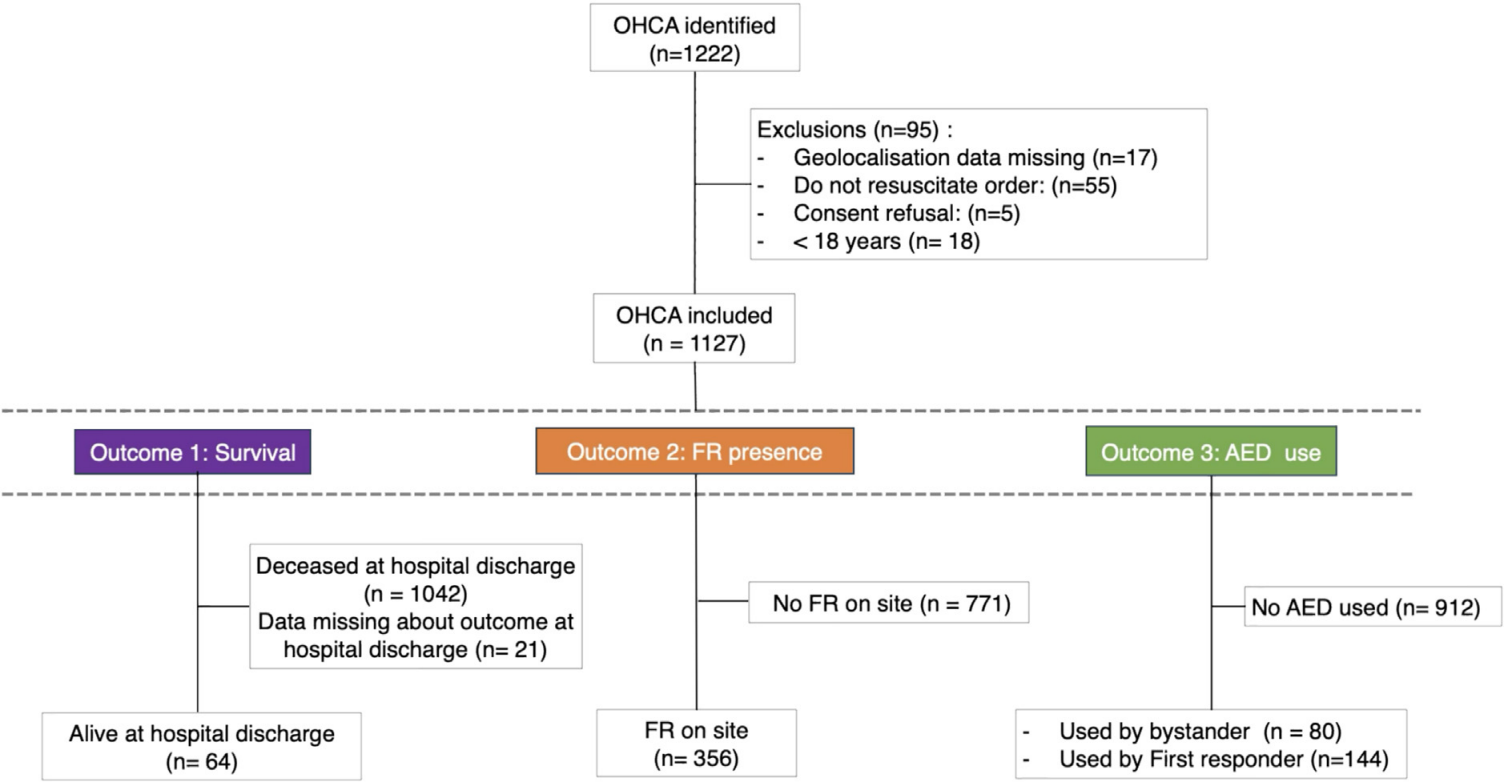
Study enrolment and distribution of the cohort for each outcome. Abbreviations: OHCA – out-of-hospital cardiac arrest; FR – first responders; AED – automated external defibrillators.

The study protocol was approved by the local ethics committee (CER-VD; ID 2024-01308). We conducted a retrospective observational study including patients who experienced an OHCA of any aetiology between January 1, 2018, and December 31, 2022, in the canton of Fribourg. Exclusion criteria were patients with a “do not resuscitate” order, those who did not consent to the use of their data, those with incomplete or incoherent data, and those <18 years old at the time of the event (Fig. 1).

### Geographical context and prehospital response system

**Fig. 2:**
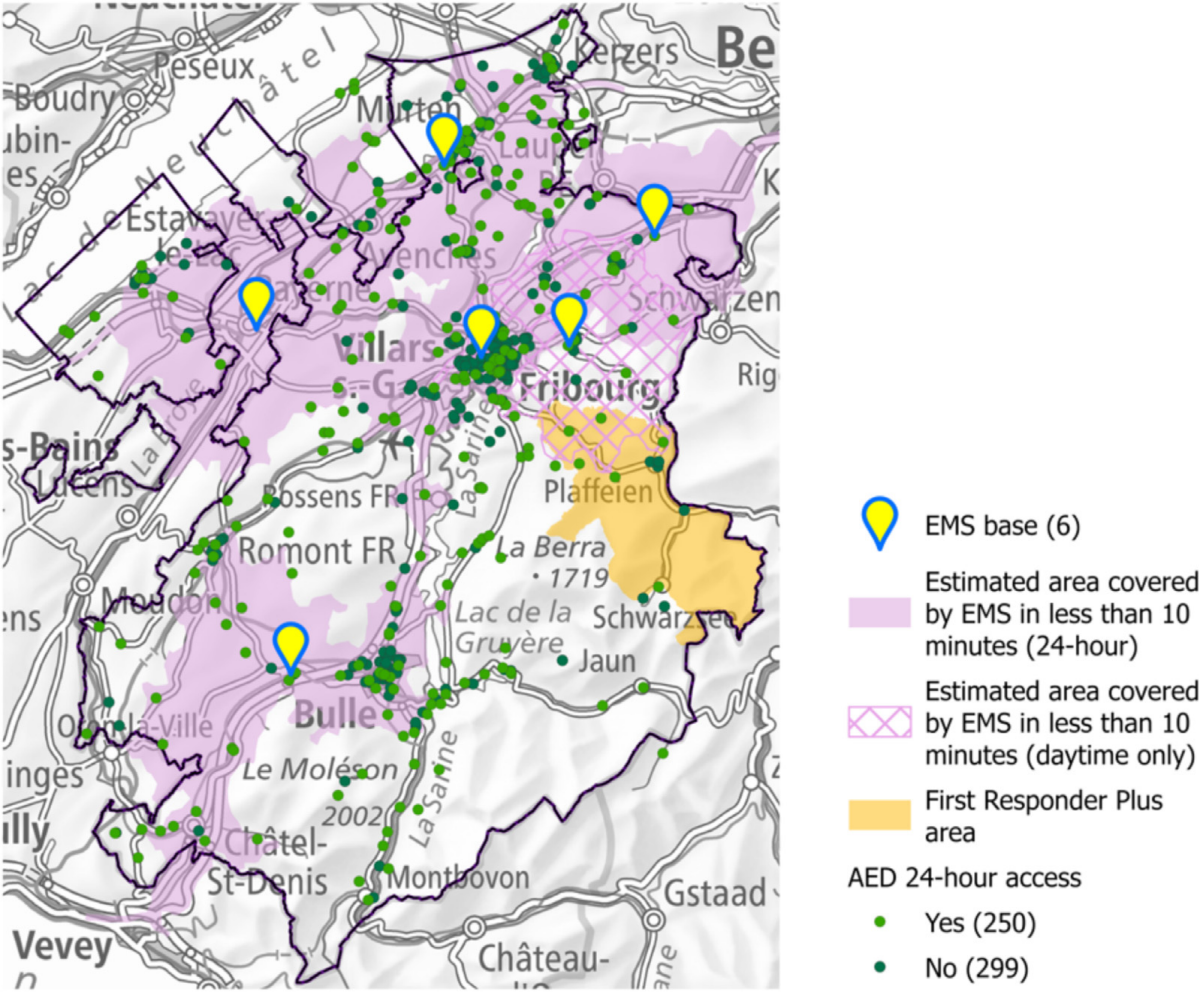
Overview of the OHCA response system, including emergency medical services’ bases, areas covered by emergency medical services in less than 10 min, first responder plus area and automated external defibrillator location according to their availability. Abbreviations: OHCA – out-of-hospital cardiac arrest; EMS – emergency medical services; AED – automated external defibrillators.

The canton of Fribourg covers an area of 1672 km^2^ in western Switzerland.^38^ In 2022, its population was 334,465 inhabitants with one-third living in three main urban areas (Fribourg, Bulle and Morat).^39^ The rest of the territory consists of peripheric and rural areas. Emergency calls are managed by a single emergency medical dispatch (EMD) centre, which coordinates all cantonal EMS. They are divided among six EMS bases and the estimated area they can cover in less than 10 min is shown in purple in Fig. 2. An emergency physician is always present in the ambulance for suspected OHCA patients. Since 2016, trained FRs may also be contacted by the EMD via a smartphone application called “Momentum”. When a suspected OHCA call is received, the dispatcher triggers an alert through the app, which geolocates nearby available FRs and notifies them. Responders can accept or decline the mission. Upon acceptance, their location is shared with the EMD, enabling coordinated guidance. The app also shows registered AED locations, allowing FRs to retrieve one on their way. Although activation occurs via the app, it is fully controlled and initiated by the EMD centre. Another association named “FR Plus” operates in the northeastern part of the canton (Fig. 2). It brings together volunteers who have received advanced training alongside paramedics, enabling them to respond not only to resuscitation cases, but also to life-threatening situations.^40^ The dispatch mode is identical for FR and FR Plus responders, both being alerted via the Momentum app by the emergency dispatch centre. Since 2016, AEDs have been registered by the EMD and integrated into the application to enable their location by FRs.

### Data definition and collection

“Location” was defined as the place where the OHCA occurred and was categorised as either “home” or “non-home”. Geographical zones were defined using two complementary approaches: territorial typology was characterized by the Swiss Federal Office of Statistics based on both morphological (population density) and functional (commuting flows) criteria and categorized as “urban”,” intermediate” or “rural” areas (“Bilan démographique selon le canton − 1971–2022 | Tableau,” 2023). Operational zones were based on EMS and FR system organization (FR Plus zone and EMS >0 min zone). The former are mutually exclusive, while the latter may overlap with each other and with the territorial zones. “Time to EMS arrival” was defined as the time gap between the call to the national emergency number (144) and arrival of the first emergency response vehicle at the patient’s location. For the remaining variables, the definitions established in the 2024 Utstein style uniform reporting guidelines were applied.

Parameters collected were: age at OHCA; gender; collapse witnessed; collapse time; 144 incoming call time; FR activation; location of the OHCA with GPS coordinates; bystander and FR CPR; bystander and FR AED use; initial cardiac rhythm; EMS CPR; Ustein-based return of spontaneous circulation (ROSC) score^41^; presence and duration of ROSC; time to EMS arrival; survival to hospital admission; survival to hospital discharge; and neurological outcome using the cerebral performance category (CPC) scale at hospital discharge. Different databases were used to collect the targeted variables and parameters. First, the SWISSRECA was used for all OHCA characteristics. Data on AED were sourced from the EMD registry, which collects information on the location, installation and availability of these devices. Information on FRs was obtained from the annual management reports.^42^ Geographical and demographical data were extracted from the Swiss Federal Office of Statistics in Neuchâtel.^43^, ^44^

### Statistical analysis

Categorical data were presented as numbers and proportions and compared using χ^2^ or Fisher’s exact tests. Means and standard deviations were used to describe continuous variables with a normal (Gaussian) distribution, while the median and interquartile range were used for non-normal distribution. Both were compared using t-tests or Mann–Whitney U tests. The distribution was analysed using the Shapiro-Wilk test. The spatial analyses involved georeferencing the locations of OHCA and AEDs, which were used to calculate outcomes at the municipal level and in strategic locations for interpretation, such as urban areas, the FR Plus area and places located outside a 10-min operational range of the EMS (Fig. 2). Isochrones were generated using openrouteservice.org with a 15-min journey from EMS bases, without considering traffic. EMS vehicles are allowed certain operational flexibilities under road traffic regulations. Estimating a 10-min travel zone, we assumed they could effectively travel up to ∼1.5 times the standard speed, though official public documentation for this threshold is limited. Heatmaps based on the kernel density method were generated (radius = 2; weight = 1) for each outcome (survival, FR presence and AED use), both with and without the criteria.^45^ Spatial analyses were conducted using ArcGIS Pro 3.1.

To account for potential confounding, we performed multivariate logistic regression. We used exact logistic regression (Firth’s penalized likelihood method) to address the rare outcome of hospital discharge (5.8 %) and potential separation issues. The analysis examined four pre-hospital interventions (bystander CPR, bystander AED use, FR CPR, FR AED use) across three outcomes: ROSC, survival at hospital admission, and survival at hospital discharge. Unadjusted models included only intervention variables, while adjusted models controlled for age, sex, EMS arrival time, initial shockable rhythm, home location, and witnessed status. Secondary analyses included geographic stratification (urban/intermediate/rural) and specific subpopulations (first responder plus zones, areas with EMS arrival >10 min). We assessed multicollinearity among continuous variables and checked for complete separation in contingency tables. Events per variable (EPV) ratios guided the appropriateness of exact regression (EPV < 10). Statistical significance was defined as *p* < 0.05, and results were reported as odds ratios (OR) with 95 % confidence intervals. Analyses were conducted with Python 3.13.0 (NumPy, pandas, rpy2 for R integration for the Firth method) in Visual Studio Code (version 1.95, © Microsoft 2025), connected to a local PostgreSQL database (version 14.15).”

## Results

### Study population

Of 1222 OHCA identified, 1127 cases were included in the final analysis (male, *n* = 802, 73 %; median age, 70 [interquartile range (IQR), 58–79] years). The study flow chart is shown in Fig. 1 and patient demographic characteristics are presented in Table 1. Most OHCA occurred at home (*n* = 795, 71 %) due to a medical cause (*n* = 897, 80 %), while 32 % (*n* = 285) took place in urban areas. Collapse was witnessed in 43 % (*n* = 486), with most incidents occurring during the day (*n* = 522,46 %) or evening (33 %). Bystander CPR was performed on 476 patients (42 %), of which 11 % (*n* = 123) were assisted by a dispatcher via phone; AED was used on 80 patients (7 %). A FR was on-site in 34 % (*n* = 356) of cases, initiated CPR in 23 % (*n* = 257), and used an AED in 13 % (*n* = 144).

**Table 1:** Characteristics of the included OHCA cases for the whole cohort and for each of the five geographical zones.

**Characteristics**	**All OHCA**	**Urban area**	**Intermediate area**	**Rural area**	**EMS >10 min**	**FR Plus**
OHCA, n (%)	1127 (100)	355 (32)	393 (35)	379 (34)	353 (31)	57 (5)
Age, median (IQR)	70 (58–79)	70 (57–80)	70 (59–79)	70 (59–79)	70 (58–78)	65 (57–75)
Male, n (%)	802 (73 %)	235 (66 %)	292 (76 %)	275 (75 %)	262 (78 %)	44 (83 %)
Home located, n (%)	795 (71 %)	250 (70 %)	275 (70 %)	270 (71 %)	251 (71 %)	38 (67 %)
Collapse witnessed, n (%)	486 (43 %)	148 (42 %)	170 (43 %)	168 (44 %)	148 (42 %)	23 (40 %)
Medical/cardiac cause, n (%)	897 (80 %)	285 (80 %)	317 (81 %)	295 (78 %)	272 (77 %)	47 (82 %)
Call time of the day, n (%)						
- Between 00:00 and 07:59	235 (21 %)	61 (17 %)	90 (23 %)	84 (22 %)	81 (23 %)	11 (19 %)
- Between 08:00 and 15:59	522 (46 %)	169 (48 %)	183 (47 %)	170 (45 %)	147 (42 %)	25 (44 %)
- Between 16:00 and 23:59	370 (33 %)	125 (35 %)	120 (31 %)	125 (33 %)	125 (35 %)	21 (37 %)
Call-assisted CPR performed, n (%)	123 (11 %)	40 (11 %)	48 (12 %)	35 (9,2%)	42 (12 %)	7 (12 %)
Bystander CPR performed, n (%)	476 (42 %)	125 (35 %)	180 (46 %)	171 (45 %)	174 (49 %)	33 (58 %)
Bystander AED used, n (%)	80 (7,1%)	22 (6,2%)	32 (8,1%)	26 (6,9%)	27 (7,6%)	5 (8,8%)
FR on-site, n (%)	356 (34 %)	73 (22 %)	153 (42 %)	130 (36 %)	151 (45 %)	39 (76 %)
FR CPR performed, n (%)	257 (23 %)	41 (12 %)	112 (28 %)	104 (27 %)	123 (35 %)	28 (49 %)
FR AED used, n (%)	144 (13 %)	22 (6,2%)	66 (17 %)	56 (15 %)	82 (23 %)	20 (35 %)
CPR performed before EMS arrival, n (%)	577 (51 %)	149 (42 %)	218 (55 %)	210 (55 %)	220 (62 %)	42 (74 %)
AED used before EMS arrival, n (%)	215 (19 %)	44 (12 %)	92 (23 %)	79 (21 %)	105 (30 %)	25 (44 %)
Shockable initial rhythm, n (%)	220 (20 %)	66 (19 %)	81 (21 %)	73 (19 %)	75 (21 %)	13 (23 %)
Time from call to EMS arrival, [minutes,] median (IQR)	12,0 (9,0–16,0)	9,0 (7,0–11,0)	13,0 (10,0–16,0)	15,0 (12,0–18,0)	16,0 (13,0–20,0)	19,0 (14,0–23,0)
No resuscitation attempted by EMS, n (%)						
- Because of other reasons	9 (0,8%)	1 (0,3%)	5 (1,3%)	3 (0,8%)	5 (1,4%)	1 (1,8%)
- Because of the Signs of Life	13 (1,2%)	2 (0,6%)	4 (1,0%)	7 (1,8%)	6 (1,7%)	2 (3,5%)
- Obviously dead	338 (30 %)	125 (35 %)	108 (27 %)	105 (28 %)	91 (26 %)	8 (14 %)
UB-ROSC derived probability of ROSC and survival to hospital admission, median (IQR)	11 (7–18)	13 (8–21)	11 (6–21)	10 (6–17)	9 (6–14)	9 (6–21)
Alive at hospital admission, n (%)	221 (20 %)	79 (22 %)	70 (18 %)	72 (19 %)	62 (18 %)	13 (23 %)
Alive at hospital discharge, n (%)	64 (5,8%)	30 (8,5%)	14 (3,6%)	20 (5,5%)	14 (4,1%)	3 (5,7%)
Missing data for outcome at hospital discharge, n (%)	21	1	7	13	15	4
CPC score at discharge, n (%)						
- CPC 1, n (%)	45 (69 %)	22 (6,1%)	11 (2,8%)	12 (3,2%)	10 (2,8%)	4 (7 %)
- CPC 2, n (%)	14 (22 %)	6 (1,7%)	1 (0,3%)	7 (1,8%)	4 (1,1%)	0 (0 %)
- CPC 3, n (%)	5 (7,7%)	2 (0,6%)	3 (0,8%)	0 (0 %)	1 (0,3%)	0 (0 %)
- Unknown, n (%)	1 (1,5%)	0 (0 %)	0 (0 %)	1 (0,3%)	0 (0 %)	0 (0 %)

Abbreviations: AED: automated external defibrillator; CPC: cerebral performance category; CPR: cardiopulmonary resuscitation; EMS: emergency medical service/s; FR: first responders; IQR: interquartile range; OHCA: out-of-hospital cardiac arrest; ROSC: return of spontaneous circulation.

Overall, CPR was provided in 51 % (*n* = 577) of OHCA cases and an AED was employed in 19 % (*n* = 215) of cases before EMS arrival. The initial cardiac rhythm was shockable in 20 % (*n* = 220) of the cohort. Median EMS arrival time was 12 min after the emergency call, while FRs arrived after 9 min. EMS did not attempt resuscitation in 31 % (*n* = 360) of cases, primarily due to obvious death (*n* = 338, 30 %) or signs of life (*n* = 13, 1,2 %). When resuscitation was attempted, the median UB-ROSC prognostic score was −19, indicating a 13 % probability of sustained ROSC and survival to hospital admission. ROSC lasted <20 min in 65 patients (6 %) and >20 min in 176 patients (16 %). 20 % (*n* = 221) patients were alive at hospital admission and 64 (5.8 %) survived to discharge, with partially missing documentation on survival (*n* = 21) or neurological outcomes (*n* = 19) at the hospital discharge. Over the surviving patients, 45 (69 %) had a cerebral performance category of 1.

### Outcomes

**Fig. 3:**
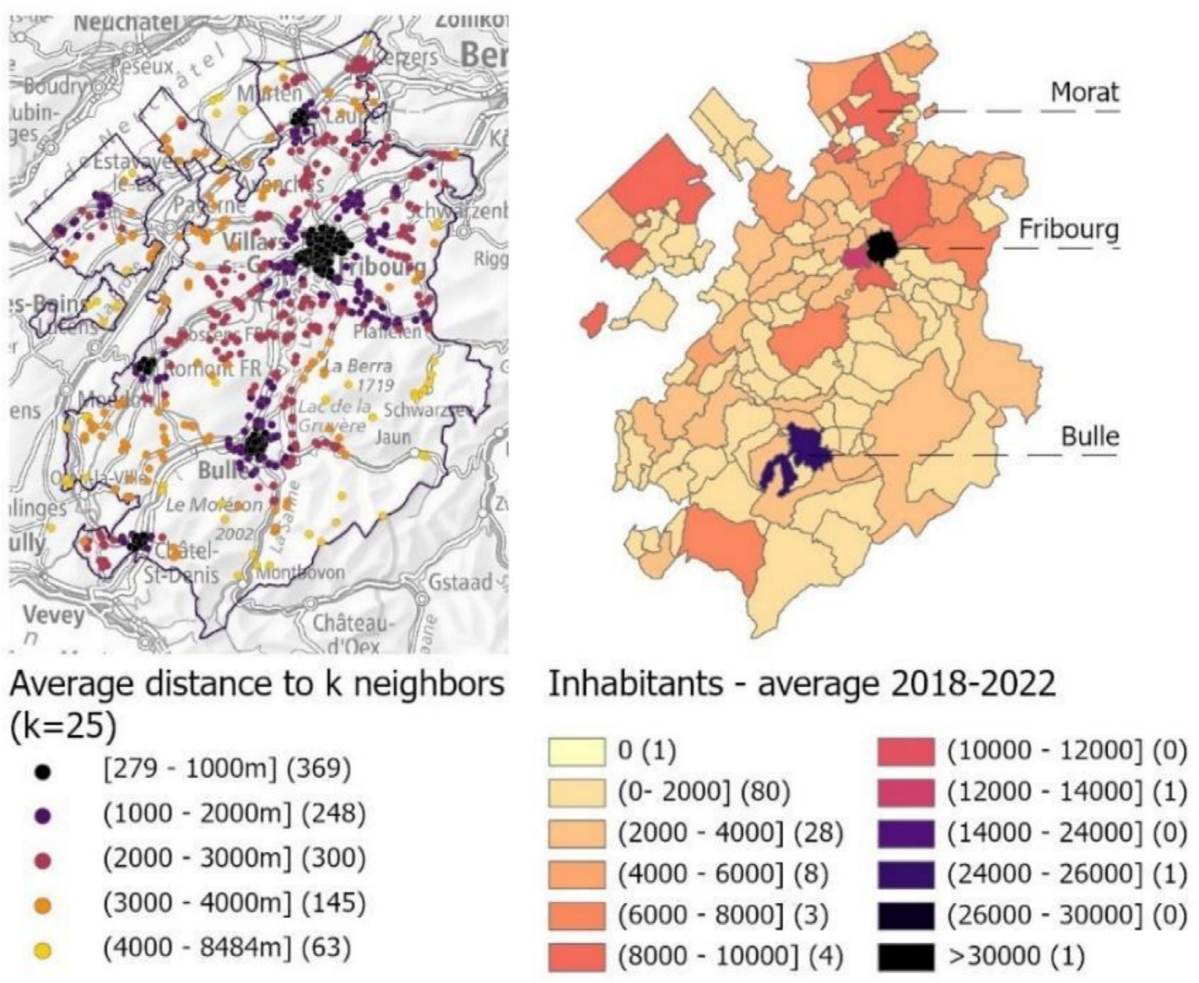
OHCA clusters and population distribution. **A (left):** map of all OHCA cases (*n* = 1127) with average Euclidean distance to *k* = 25 nearest neighbours*.***B (right):** map of the resident population by municipality, based on the average number of inhabitants from 2018 to 2022. Abbreviations: OHCA – out-of-hospital cardiac arrest.

The outcomes of the primary aim represented by the OHCA clusters compared to the population distribution across the territory are shown in Fig. 3**a-b**. The heatmap of all OHCA (*n* = 1127) showed that the highest densities were observed around the main cities of Fribourg, Bulle and Morat, which correspond to the most populated areas of the canton. Fig. A1-2 provides a detailed visualization of population distribution and absolute OHCA occurrences.

**Fig. 4:**
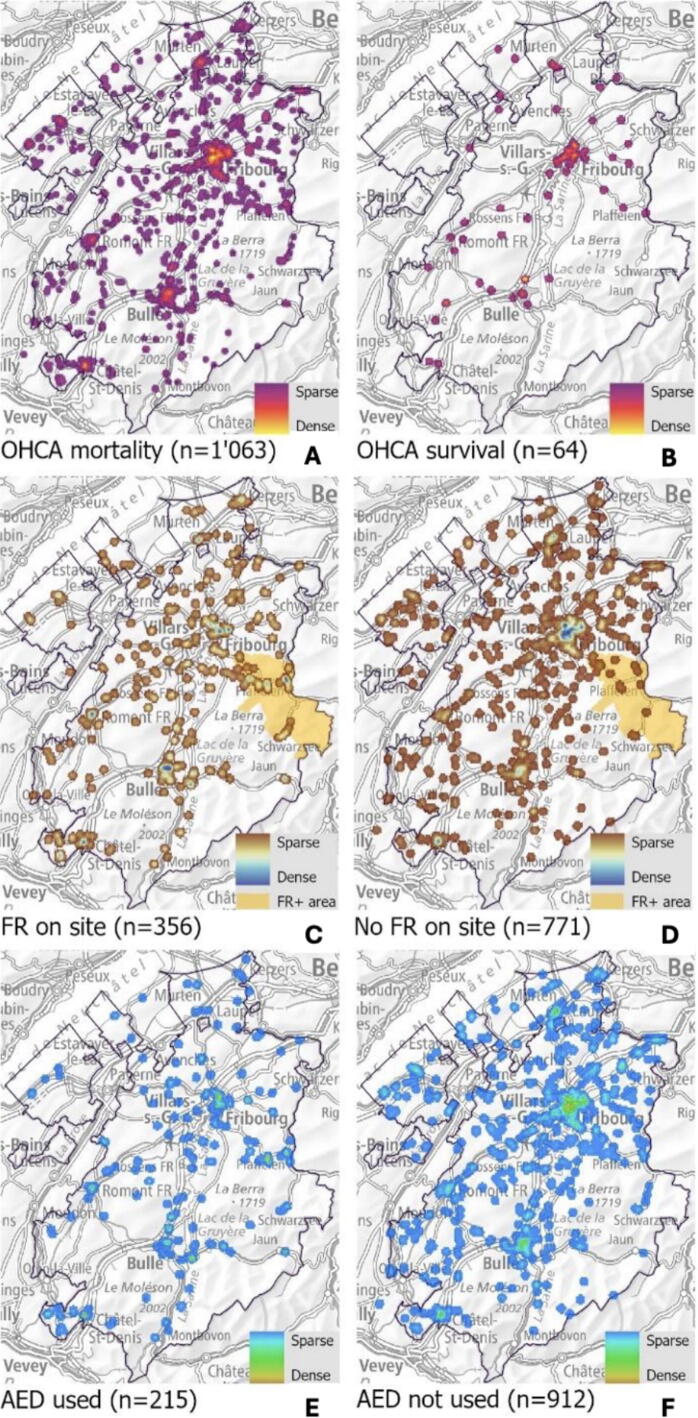
Heatmaps illustrating OHCA clusters with a focus on survival at hospital discharge, presence of first responders and automated external defibrillator use. **Top row**. Comparison of OHCA based on patient outcome**. A** (left) shows cases that resulted in “mortality” (n=1063). **B** (right) shows “survival” cases (*n* = 64). **Middle row.** Comparison of OHCA based on whether **C** (left) a FR was “present on-site” (*n* = 356) or **D** (right) “not present on site” (*n* = 771). The yellow area represents the zone where FRs plus are active. **Bottom row**. Comparison of OHCA based on whether **E** (left) an “automated external defibrillator was used” (*n* = 215) or **F** (right) “not used” (*n* = 912). Abbreviations: OHCA – out-of-hospital cardiac arrest; FR – first responders; AED – automated external defibrillators.

The secondary aim used heatmaps of “OHCA survival”, “FR presence” and “AED use” as outcomes (Fig. 4**a-f**). Mortality clusters (Fig. 4**a**) were correlated to the occurrence of cases (Fig. 3**a**). No clusters were observed outside densely populated areas (Fig. 3**b**). Survivors were spread throughout the territory, with signs of spatial grouping in the cities of Fribourg, Bulle and Morat (Fig. 4**b**).

Regarding the presence of FRs during OHCA incidents (Fig. 4**c**), their spatial distribution did not strictly follow occurrence density (Fig. 3**a**) but showed a more even spread across the territory. Clusters were concentrated in major cities with notable differences. Fribourg and Morat showed a larger absence of clusters (Fig. 4**d**), while Bulle, Châtel-St-Denis and the FR Plus area exhibited a greater FR presence (Fig. 4**c**). Overall, the northern and western parts of the canton have fewer FRs than the southern and eastern areas. The FR percentage on an OHCA site is represented in supplementary materials (Fig. B1). For AED use during OHCA incidents, no clear clusters were formed, with cases scattered across the territory. However, slight signs of aggregation were visible in the cities of Fribourg and Bulle, as well as in the FR Plus area (Fig. 4**e**). For OHCA cases without AED use (Fig. 4**f**), clusters corresponded to the population distribution and OHCA occurrence. The number of OHCA considered covered, i.e. an AED was available within 100 m, was 125 (11 %), of which 53 (5 %) were in an urban area. The number of AED per 10,000 inhabitants, as well as the nearest AED to each OHCA are mapped in Fig. C1-2. The overall evolution of these secondary outcomes (survival, FR and AED) over the five-year period is shown in Fig. D. In 2018, 1850 FRs and 326 AED were registered in the canton. By the end of 2022, the numbers had increased to 2050 FRs and 549 AED, with 250 (51 %) available 24/7.

The tertiary aims focused on the proportion of the secondary outcomes in the five distinct strategical geographical areas (urban, intermediate, rural, FR plus and EMS >10-min zones; Fig. 2). Detailed results for survival, FR presence and AED use are reported in Table 2.

**Table 2:** Principal outcomes over the five geographical zones.

	**All OHCA** **(*n* = 1097)**	**Urban area** **(*n* = 350)**	**Intermediate area** **(*n* = 378)**	**Rural area** **(*n* = 369)**	**EMS** **> 10 min** **(*n* = 344)**	**FR Plus** **(*n* = 55)**
Survival, n (%)	64 (5,8%)	30 (8,5%)	14 (3,6%)	20 (5,5%)	14 (4,1%)	3 (5,7%)
FR on-site, n (%)	356 (34 %)	73 (22 %)	153 (42 %)	130 (36 %)	151 (45 %)	39 (76 %)
AED used before EMS arrival, n (%)	215 (19 %)	44 (12 %)	92 (23 %)	79 (21 %)	105 (30 %)	25 (44 %)

Abbreviations: AED: automated external defibrillator; EMS: emergency medical service/s; FR: first responders; OHCA: out-of-hospital cardiac arrest; SCF = Swiss Canton Fribourg.

Survival rates were low (≤8.5 %) across all areas with the highest rate observed in urban areas and lowest rate in intermediate (3.6 %) areas and EMS response times of >10 min areas (4.1 %), highlighting the critical impact of a delayed response. Survival in the FR plus area was 5.7 % with an overall survival rate of 5.8 % in the canton underscoring the challenges of OHCA management.

The presence of FRs varied considerably with 69 % (*n* = 38) in the FR Plus area, indicating the strongest cantonal FR deployment/activation. Across all OHCA cases, there was a FR presence of 31 %, showing that FRs are absent in most cases. In urban areas, only 21 % (*n* = 73) FRs were present despite a high population density (Fig. 3**b, A1**). In areas with an EMS travel time >10 min, FRs were present in 31 % of OHCA occurrences. Fig. B1 provides a more detailed FR presence at district level.

In unadjusted analyses, bystander CPR was strongly associated with increased survival (OR = 2.49, 95 % CI: 1.44–4.33, *p* = 0.001), but this association was attenuated after adjustment for patient and event characteristics (adjusted OR = 1.19, 95 % CI: 0.62–2.28, *p* = 0.594). FR CPR was associated with significantly lower odds of survival both before and after adjustment, including geographic variables (OR = 0.26, 95 % CI: 0.05–0.89, *p* = 0.029). FR AED use showed a non-significant trend toward improved survival (adjusted OR = 2.53, 95 % CI: 0.59–14.71, *p* = 0.219). Among other covariates, shockable initial rhythm (OR = 8.80, 95 % CI: 4.67–17.22, *p* < 0.001), witnessed arrest (OR = 6.32, 95 % CI: 2.96–15.41, *p* < 0.001), and shorter EMS response time (OR per min = 0.89, 95 % CI: 0.82–0.95, *p* < 0.001) were the strongest predictors of survival. Arrests at home were associated with decreased odds of survival (OR = 0.47, 95 % CI: 0.25–0.89, *p* = 0.022), while geographic zone type was not significantly associated with outcomes (*p* = 0.172). Geographic subgroup analyses did not reveal statistically significant associations. In urban areas, bystander CPR showed a strong unadjusted effect (OR = 3.60, 95 % CI: 1.61–8.26, *p* = 0.002), attenuated in adjusted models (OR = 1.69, *p* = 0.317). In rural and intermediate zones, the effect of bystander interventions was weaker and not statistically significant. Across all areas, FR CPR was associated with either neutral or negative outcomes, including in urban settings (adjusted OR = 0.08, 95 % CI: 0.00–1.00, *p* = 0.050). Analyses within the first responder plus zones (*n* = 53) were underpowered and no significant associations were observed. Trends were consistent with the main analysis: a positive effect of bystander CPR, a negative effect of bystander AED use, and a negative effect of FR CPR.

## Discussion

This study provides the first comprehensive geographical evaluation of the OHCA response system in the canton of Fribourg. Mapping of the distribution of OHCA and analysing resources such as FR presence and AED use across different regions allowed to highlight significant disparities and opportunities for improvement. Epidemiological characteristics of OHCA in the canton were similar to other national^26^, ^34^, ^35^, ^46^ and international^47^ cohorts. In general, most patients are men in their seventies, the event typically occurs at home without a witness, and most often during the day or evening^48^ with initial non-shockable rhythm. It is important to consider those factors as well as the circumstances of the event,^41^, ^49^ as they can influence the geographical distribution of survival zones.^27^ As expected, the density pattern of OHCA cases followed population density. Clusters of survival to hospital discharge were identified in urban areas, similar to a study in the canton of Vaud.^35^ With a main cluster in the main city, our results align with those reported in a French study, which demonstrated better survival in areas close to ambulance centres and specifically where physicians could provide advanced cardiac life support.^21^

Concerning FRs, several disparities were revealed by spatial analysis. While urban areas enjoy the highest survival rates, they have relatively lower levels of FR presence. Notably, the canton of Luzern obtained comparable results with a similar methodological approach.^34^ The latest OHCA study in our canton demonstrated that the sequential activation of the EMS led to a delayed arrival of FRs.^37^ However, the actual distance travelled to retrieve an AED was not measured, and arrival time of FR was recorded retrospectively, which may introduce errors. In contrast, the FR Plus zone, benefitted from the highest presence of FRs. In other studies, these advanced FRs are often medical professionals and are not spread across a geographical zone.^50^ Finally, the cantonal FR network enhances the EMS system by ensuring a stronger presence in areas located >10 min away from an EMS intervention, a critical factor for improving OHCA outcomes.^51^ These regional differences offer an opportunity to tailor recruitment efforts at the municipality level to complement the insufficient FR presence. This study also highlights the effectiveness of a small-scale, advanced FR system in urban and non-urban areas, providing a potential blueprint for other national or international regions facing similar challenges, particularly those distant from EMS bases.

AEDs were used throughout the territory with a very low proportion in urban areas and a higher use in areas with limited EMS access. Use rates varied greatly by region: AEDs were deployed in 44 % (*n* = 25) of FR Plus cases and in 30 % (*n* = 105) of arrests occurring in areas where EMS travel time exceeded 10 min, highlighting their crucial role in delayed-response settings. In contrast, AED use in urban zones was 12 % (*n* = 44), despite high device density (Fig. 4f), most likely reflecting shorter EMS arrival times. A discrepancy between potential availability and actual use has been demonstrated in Copenhagen.^52^ In our cohort, overall AED use was 19 % (*n* = 215), indicating room for improvement. In contrast, we observed a higher proportion of use (19 %) than the theoretical coverage of a 100-metre radius (11 %), probably because usage is higher in non-urban areas where FRs mostly travel by bike or car.^32^ The cohort was evenly distributed across geographic areas: urban (32.0 %), intermediate (34.9 %), and rural (33.2 %). Although FRs and AEDs are broadly distributed across the canton, their density remains insufficient to ensure adequate coverage, particularly in less populated or hard-to-reach areas. In 2022, there were 2050 registered FRs and 549 AEDs in the canton, while the canton of Ticino had three times as many with nearly the same population.^30^ The cantonal AED density per km^2^ is also lower than reported in comparable national and international studies^46^, ^47^, ^48^ and well below European guidelines.^7^ While the number of registered FRs has stagnated during the study period, the number of AEDs has nearly doubled. It is therefore necessary to increase FR registrations and further expand AED coverage.

Our findings are in line with recent international literature underlining both the potential and limitations of FR systems in rural areas. For example, although FRs in rural Sweden often arrive before EMS, survival does not necessarily improve due to factors such as unwitnessed collapses and delays in recognition or activation.^53^ Similarly, in our cohort, higher FR presence and AED use in the rural and FR Plus zones did not translate into higher survival, suggesting that FR activity alone may not overcome the survival disadvantage of prolonged EMS travel times. Furthermore, geospatial optimization for AED deployment is primordial in low-density areas. In our study, AED use was particularly high in remote zones with better FR coverage, supporting the strategy of equipping FRs with mobile AEDs. These findings emphasize the importance of combining geospatially-informed AED placement with targeted FR deployment to improve equity in OHCA outcomes.^54^

The main strength of this study is its comprehensive analysis of an OHCA management system at the cantonal level. Its geographical perspective offers valuable insights into a topic that has rarely been examined from this angle. The study also benefits from the use of the SWISSRECA database allowing national and international comparison. Finally, the FR Plus zone may serve as a model for others to enable strategic FR deployment. Our study has some limitations. First, the retrospective analysis depended on routinely collected data from multiple contributors, leading to a variability in completeness and quality. A significant challenge was the absence of standardised protocols for paramedics when completing intervention forms, a short coming also observed in international OHCA datasets.^55^ In addition, Euclidean (as the crow flies) distances to the nearest AED were calculated without accounting for buildings or terrain, a method consistent with previous studies and allowing for comparability.^16^ Clusters were identified descriptively based on visual patterns observed in the spatial maps, without a predefined algorithmic or statistical threshold. This approach, while useful for highlighting geographic trends, introduces an element of subjectivity. Furthermore, SWISSRECA is based on the 2015 Utstein template ^56^ as the 2024 version was not published at the time of the study.^57^ Some core elements from the recently published study reporting standard of FR and AED networks were also not accessible at the time of data collection.^58^ Finally, while some findings may be applicable to comparable regions, caution should be exercised when extrapolating results. Differences in geography, infrastructure, and demographic profiles across cantons and internationally can significantly influence both EMS performance and community response dynamics.

## Conclusion

This study shows that the prehospital response system for OHCA in the canton of Fribourg is geographically well-distributed and complements the EMS network. Higher proportions of FR presence and AED use in areas with delayed EMS access highlight the system's adaptability to rural needs. Bystander CPR was associated with improved survival, though this effect diminished after adjustment. First responder CPR was linked to lower odds of survival, while first responder AED use showed a non-significant trend toward benefit. Shockable rhythm, witnessed arrest, and shorter EMS response time remained the strongest predictors of survival. However, stagnation in FR recruitment and disparities in AED coverage emphasise the need for targeted efforts to increase FR registrations and expand AED deployment. Geospatial analyses provide valuable insights to guide the optimization of future resources and improve canton-wide survival outcomes.

## CRediT authorship contribution statement

**Cynthia Gay:** Writing – review & editing, Writing – original draft, Visualization, Software, Resources, Methodology, Investigation, Formal analysis, Data curation, Conceptualization. **Ludovic Galofaro:** Writing – review & editing, Writing – original draft, Visualization, Resources, Project administration, Methodology, Investigation, Formal analysis, Data curation, Conceptualization. **Théophile Emmanouilidis:** Writing – review & editing, Writing – original draft, Visualization, Software, Resources, Methodology, Formal analysis, Data curation, Conceptualization. **Diane Blaser:** Writing – review & editing, Project administration. **Sébastien Pugnale:** Writing – review & editing, Methodology, Formal analysis, Data curation. **Dorian Garin:** Software, Formal analysis. **Alexis Cogne:** Formal analysis, Data curation. **Vincent Ribordy:** Writing – review & editing, Validation, Resources, Project administration, Funding acquisition, Conceptualization. **Youcef Guechi:** Writing – review & editing, Validation, Supervision, Methodology, Investigation, Conceptualization.

## Funding

This research did not receive any specific grant from funding agencies in the public, commercial, or not-for-profit sectors.

## Declaration of competing interest

The authors declare that they have no known competing financial interests or personal relationships that could have appeared to influence the work reported in this paper.
