# Supplementary material for: Geospatial mapping of disparities in out-of-hospital cardiac arrests in the Swiss canton of Fribourg, 2018–2022: A retrospective observational study
Source: Resusc Plus. 2025 Aug 23;26:101075. doi: 10.1016/j.resplu.2025.101075 (PMC12446618; doi:10.1016/j.resplu.2025.101075)

## **Appendices**

**Figure A1.** OHCA incidents per municipality.
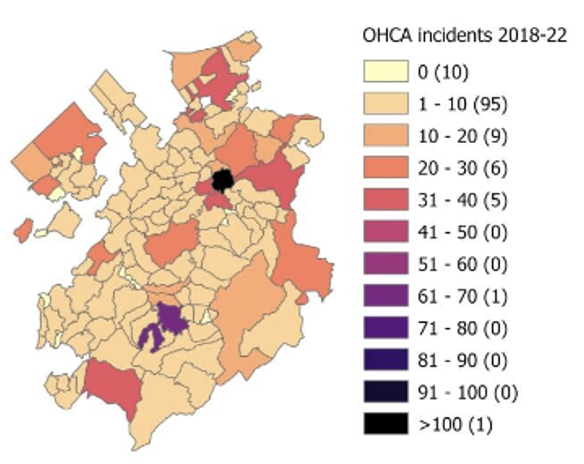


This map illustrates the distribution of OHCA incidents across different municipalities of Fribourg between 2018 and 2022. Each area is color-coded, based on the number of incidents reported, ranging from pale yellow (0 incidents) to deep purple (>100 incidents). Most regions recorded fewer than 20 incidents (represented in light colors), while only a small number of areas reported over 50 incidents. The black and dark purple areas highlight the regions with the highest concentration of incidents (>100 and 71–80, respectively). The numbers in parentheses within the legend indicate the count of regions falling under each category.

*Abbreviations*: OHCA – out-of-hospital cardiac arrest

**Figure A2.** OHCA incidence per 100,000 inhabitants.


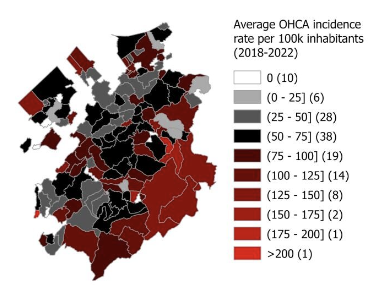


This map shows the average incidence rate of OHCA per 100,000 inhabitants from 2018 to 2022 across the municipalities. These regions are color-coded, with black and white shades indicating lower incidence rates (0–75), while reddish shades highlight higher incidence rates (75–200+). The deepest red indicates the highest rate of over 200 incidents per 100,000 inhabitants, observed in only one region. The numbers in parentheses in the legend represent the count of regions within each incidence rate category.

*Abbreviations*: OHCA – out-of-hospital cardiac arrest

**Figure B1.** First responders on site during OHCA occurrence.


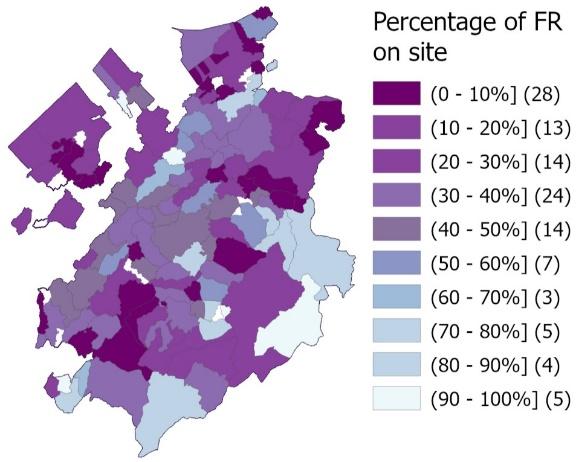


This map displays the percentage of first responders (FR) arriving on-site for incidents across the municipalities. These regions are shaded in varying colors, from dark purple (0–10%) to light blue (90–100%), representing the proportion of incidents where a FR was present. Most municipalities fall within the lower categories (0–40%), as indicated by the darker shades, while only a few areas have higher percentages of FR presence (70–100%). The numbers in parentheses within the legend show the count of regions corresponding to each percentage range.

*Abbreviations*: OHCA – out-of-hospital cardiac arrest, FR – first responders

**Figure C1.** Number of automated external defibrillators per 10,000 inhabitants by municipality


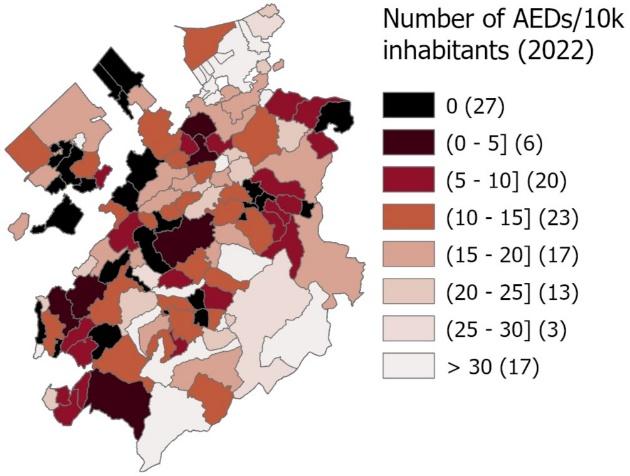


This map shows the distribution of automated external defibrillators (AED) per 10,000 inhabitants by municipalities in 2022. These regions are color-coded, with black indicating areas with no AED (0) and lighter shades representing higher densities of AED, up to over 30 per 10,000 inhabitants. Most regions have fewer than 15 AED per 10,000 inhabitants, while a few regions, depicted in the lightest shades, have more than 30 AEDs per 10,000 inhabitants. The numbers in parentheses in the legend indicate the count of regions in each AED density category.

**Figure C2.** Distance to the nearest automated external defibrillator at the time of the OHCA.


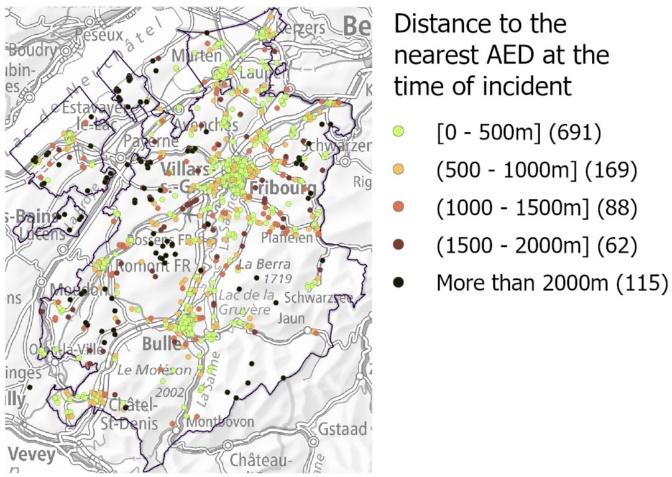


This map illustrates the distance to the nearest automated external defibrillator (AED) at the time of an incident. Each point represents an OHCA and is color-coded by the distance to the closest AED, ranging from green (0–500 m) to black (>2 km). Most incidents (691 cases) occurred within 500 m of an AED, as indicated by the abundance of green points. However, a significant number of incidents (115 cases, shown in black) occurred at distances greater than 2 km from an AED, highlighting areas with lower AED accessibility. The legend includes the number of incidents per distance category.

*Abbreviations*: OHCA – out-of-hospital cardiac arrest

**Figure D.** Evolution of proportion of survival at hospital discharge, first responder presence on site and availability of an automated external defibrillator between 2018 and 2022.


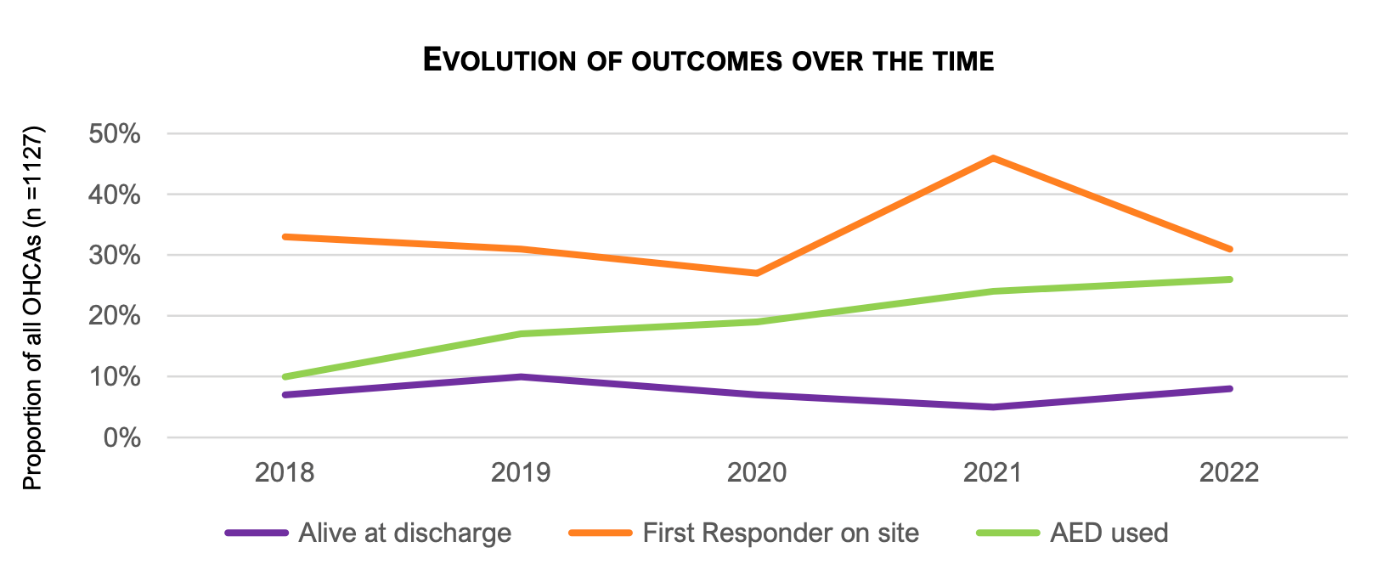


This graph depicts the evolution of outcomes for OHCA from 2018 to 2022, based on a total of 1127 cases. It tracks the following three key proportions.

1. **Alive at discharge** (purple): the percentage of patients who survived to discharge remained relatively stable over the years, with a slight increase towards the end of the period.
2. **First responder (FR) on-site** (orange): this proportion peaked in 2021 at approximately 40% but declined after.
3. **Automated external defibrillator (AED)** **used** (green): the use of AED showed a gradual increase over time, indicating improved AED accessibility or utilization. In 2018, 1850 FRs and 326 AEDs were registered in the canton. By the end of 2022, the numbers had increased to 2050 FRs and 549 AEDs, with 250 (51%) available 24/7. The others are in buildings with limited or unspecified opening hours.

*Abbreviations*: OHCA – out-of-hospital cardiac arrest

**Table 3.1. Pre-hospital interventions and survival to hospital discharge in out-of-hospital cardiac arrest (All patients, unadjusted and adjusted)**

| **Variable** | **OR** | **[95% CI]** | **p-value** |
| --- | --- | --- | --- |
| **unadjusted** | | | |
| **No intervention (Reference)** | 1.00 | __ | __ |
| **Bystander CPR** | 2.49 | [1.44-4.33] | 0.001 |
| **Bystander AED** | 1.19 | [0.50-2.56] | 0.670 |
| **First responder CPR** | 0.27 | [0.06-0.08] | 0.014 |
| **First responder AED** | 2.34 | [0.64-10.94] | 0.207 |
| **adjusted** | | | |
| **Bystander CPR** | 1.19 | 0.62–2.27 | 0.602 |
| **Bystander AED** | 0.87 | 0.31–2.25 | 0.782 |
| **First responder CPR** | 0.26 | 0.05–0.89 | 0.029 |
| **First responder AED** | 2.53 | 0.59–14.71 | 0.219 |
| **Age (per year)** | 1.00 | 0.98–1.02 | 0.779 |
| **Male sex** | 0.89 | 0.45–1.84 | 0.749 |
| **Time to EMS arrival (per min)** | 0.89 | 0.82–0.95 | <0.001 |
| **Shockable initial rhythm** | 8.80 | 4.67–17.22 | <0.001 |
| **Home location** | 0.47 | 0.25–0.89 | 0.022 |
| **Witnessed arrest** | 6.32 | 2.96–15.41 | <0.001 |
| **Geographic type** | 1.33 | 0.88–2.00 | 0.172 |

Abbreviations: CPR – cardiopulmonary resuscitation; AED – automated external defibrillator; OR – odds ratio; CI – confidence interval; EMS – emergency medical services; FR – first responders.

**Table 3.2. Pre-hospital interventions and survival to hospital discharge in out-of-hospital cardiac arrest (All patients, adjusted, without geographic type)**

| **Variable** | **OR** | **[95% CI]** | **p-value** |
| --- | --- | --- | --- |
| **No intervention (Reference)** | 1.00 | [0.62–2.28] | __ |
| **Bystander CPR** | 1.19 | [0.33–2.32] | 0.001 |
| **Bystander AED** | 0.90 | [0.05–0.95] | 0.670 |
| **First responder CPR** | 0.28 | [0.57–14.09] | 0.014 |
| **First responder AED** | 2.44 | [0.98–1.02] | 0.207 |
| **Age (per year)** | 1.00 | [0.47–1.89] | 0.776 |
| **Male sex** | 0.92 | [0.85–0.96] | 0.807 |
| **Time to EMS arrival (per min)** | 0.90 | [0.62–2.28] | 0.001 |
| **Shockable initial rhythm** | 8.31 | [4.45–16.06] | <0.001 |
| **Home location** | 0.48 | [0.25–0.91] | 0.025 |
| **Witnessed arrest** | 6.17 | [2.88–15.03] | <0.001 |

**Table 4.1. Geographic subgroup analysis of pre-hospital interventions and survival – Urban area**

| **Variable** | **OR [95% CI]** | **p-value** | **OR [95% CI]** | **p-value** |
| --- | --- | --- | --- | --- |
|  | **Unadjusted** | | **Adjusted** | |
| **No intervention (Reference)** | 1.00 | _ | 1.00 | _ |
| **Bystander CPR** | 3.60 [1.61–8.26] | 0.602 | 1.69 [0.60–4.74] | 0.317 |
| **Bystander AED** | 1.21 [0.34–3.68] | 0.782 | 1.33 [0.27–6.28] | 0.714 |
| **First responder CPR** | 0.19 [0.00–1.52] | 0.029 | 0.08 [0.00–1.00] | 0.050 |
| **First responder AED** | 1.00[0.01–190.95] | 0.219 | 1.41[0.01-345.79] | 0.877 |

**Table 4.2. Geographic subgroup analysis of pre-hospital interventions and survival – Intermediate area**

| **Variable** | **OR [95% CI]** | **p-value** | **OR [95% CI]** | **p-value** |
| --- | --- | --- | --- | --- |
|  | **Unadjusted** | | **Adjusted** | |
| **No intervention (Reference)** | 1.00 | _ | 1.00 | _ |
| **Bystander CPR** | 3.48 [1.12–12.45] | 0.032 | 1.88 [0.47–8.69] | 0.375 |
| **Bystander AED** | 0.67 [0.07–3.08] | 0.641 | 0.42 [0.04–2.40] | 0.358 |
| **First responder CPR** | 0.58 [0.07–2.61] | 0.515 | 0.58 [0.05–3.27] | 0.568 |
| **First responder AED** | 1.64 [0.26–14.32] | 0.603 | 1.43 [0.18–19.50] | 0.748 |

**Table 4.3. Geographic subgroup analysis of pre-hospital interventions and survival – Rural area**

| **Variable** | **OR [95% CI]** | **p-value** | **OR [95% CI]** | **p-value** |
| --- | --- | --- | --- | --- |
|  | **Unadjusted** | | **Adjusted** | |
| **No intervention (Reference)** | 1.00 | _ | 1.00 | _ |
| **Bystander CPR** | 1.16 [0.42–3.05] | 0.768 | 0.48 [0.14–1.53] | 0.217 |
| **Bystander AED** | 2.42 [0.56–8.71] | 0.219 | 2.66 [0.40–17.48] | 0.306 |
| **First responder CPR** | 0.53 [0.06–2.32] | 0.442 | 0.53 [0.04–3.49] | 0.541 |
| **First responder AED** | 2.68 [0.47–27.48] | 0.279 | 2.48 [0.31–33.01] | 0.407 |

**Table 4.4. Geographic subgroup analysis of pre-hospital interventions and survival – FR plus area**

| **Variable** | **OR [95% CI]** | **p-value** | **OR [95% CI]** | **p-value** |
| --- | --- | --- | --- | --- |
|  | **Unadjusted** | | **Adjusted** | |
| **No intervention (Reference)** | 1.00 | _ | 1.00 | _ |
| **Bystander CPR** | 1.37 [0.17–15.80] | 0.765 | 0.70 [0.00–47.33) | 0.832 |
| **Bystander AED** | 1.34 [0.01–35.93] | 0.868 | 7.98 [0.01–34900.91] | 0.451 |
| **First responder CPR** | 0.84 [0.03–16.30] | 0.909 | 1.99 [0.00–337.02] | 0.749 |
| **First responder AED** | 2.51 [0.18–92.61] | 0.527 | 1.16 [0.01–441.73] | 0.936 |

**Table 4.5. Geographic subgroup analysis of pre-hospital interventions and survival – EMS > 10 min zone**

| **Variable** | **OR [95% CI]** | **p-value** | **OR [95% CI]** | **p-value** |
| --- | --- | --- | --- | --- |
|  | **Unadjusted** | | **Adjusted** | |
| **No intervention (Reference)** | 1.00 | _ | 1.00 | _ |
| **Bystander CPR** | 2.40 [0.74–8.75] | 0.146 | 0.67 [0.15–2.99] | 0.598 |
| **Bystander AED** | 2.39 [0.54–8.75] | 0.230 | 1.42 [0.16–11.34] | 0.751 |
| **First responder CPR** | 0.61 [0.07–2.84] | 0.566 | 0.31 [0.02–2.21] | 0.264 |
| **First responder AED** | 1.34 [0.21–12.68] | 0.763 | 1.87 [0.21–30.63] | 0.589 |

*Abbreviations: CPR: Cardiopulmonary resuscitation; AED: Automated external defibrillator; OR: Odds ratio; CI: Confidence interval; EMS: Emergency medical services; FR: First responder.*

*Adjusted models control for age, sex, witnessed arrest, initial rhythm, location, and time to EMS arrival*

*Geographic model additionally adjusts for geographic-specific factors*

**OHCA Fribourg Website Spatial Analysis:**

**https://maps.fr.ch/portal/apps/instant/sidebar/index.html?appid=d89bc4b59609450186dbe9415c08c253**

& QR Code:


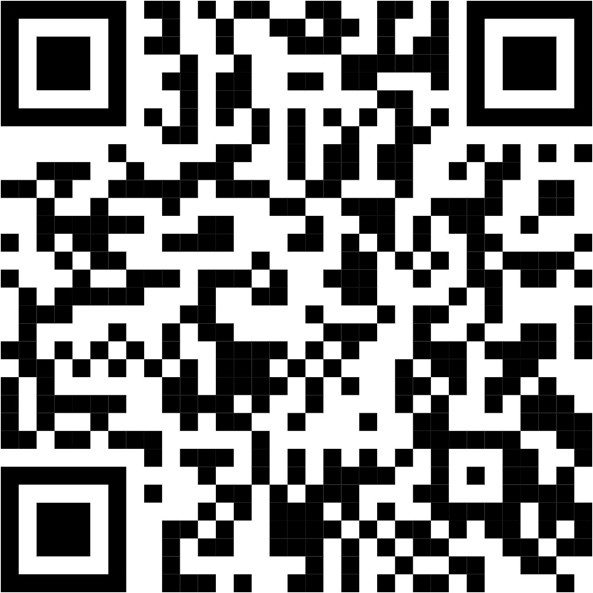

Supplement: Supplementary Data 1 [file mmc1.docx]
